# Mitochondrial Dysfunction in Heart Failure: From Pathophysiological Mechanisms to Therapeutic Opportunities

**DOI:** 10.3390/ijms25052667

**Published:** 2024-02-25

**Authors:** Giovanna Gallo, Speranza Rubattu, Massimo Volpe

**Affiliations:** 1 Department of Clinical and Molecular Medicine, Sapienza University of Rome, Via di Grottarossa 1035-1039, 00189 Rome, RM, Italy; giovanna.gallo@uniroma1.it; 2 IRCCS Neuromed, 86077 Pozzilli, IS, Italy; 3 IRCCS San Raffaele Roma, 00166 Rome, RM, Italy; massimo.volpe@sanraffaele.it

**Keywords:** mitochondria, electron transport chain, oxidative stress, inflammation, heart failure, cardiac disease, cellular recovery, cardiac rehabilitation

## Abstract

Mitochondrial dysfunction, a feature of heart failure, leads to a progressive decline in bioenergetic reserve capacity, consisting in a shift of energy production from mitochondrial fatty acid oxidation to glycolytic pathways. This adaptive process of cardiomyocytes does not represent an effective strategy to increase the energy supply and to restore the energy homeostasis in heart failure, thus contributing to a vicious circle and to disease progression. The increased oxidative stress causes cardiomyocyte apoptosis, dysregulation of calcium homeostasis, damage of proteins and lipids, leakage of mitochondrial DNA, and inflammatory responses, finally stimulating different signaling pathways which lead to cardiac remodeling and failure. Furthermore, the parallel neurohormonal dysregulation with angiotensin II, endothelin-1, and sympatho-adrenergic overactivation, which occurs in heart failure, stimulates ventricular cardiomyocyte hypertrophy and aggravates the cellular damage. In this review, we will discuss the pathophysiological mechanisms related to mitochondrial dysfunction, which are mainly dependent on increased oxidative stress and perturbation of the dynamics of membrane potential and are associated with heart failure development and progression. We will also provide an overview of the potential implication of mitochondria as an attractive therapeutic target in the management and recovery process in heart failure.

## 1. Introduction

The heart is an organ with high energy demand which contains, at the cardiomyocyte level, an elevated concentration of mitochondria that are responsible for oxidative metabolism and the majority (95%) of ATP production [1]. Mitochondrial dysfunction occurs in patients with heart failure (HF) and is associated with a maladaptive response and a progressive decline in bioenergetic reserve capacity irrespective of the etiology of the disease [2]. Neurohormonal dysfunction, systemic inflammation, and cell stress contribute to mitochondrial dysfunction in HF, with a consequent increase in reactive oxygen species (ROS) production, nitric oxide synthase (NOS) uncoupling, and altered cellular calcium regulation. These molecular mechanisms contribute to cellular apoptosis, fibrosis, and cardiomyocyte hypertrophy finally leading to cardiac remodeling and HF development [3,4,5].

**Figure 1:**
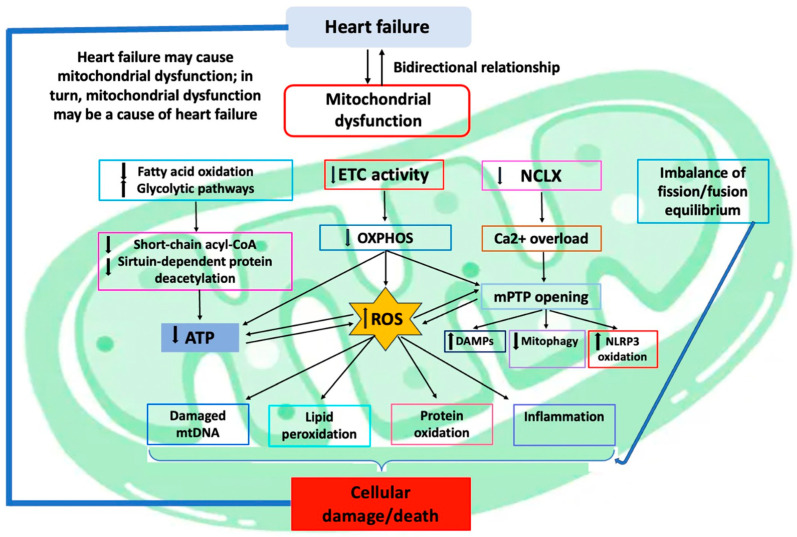
Pathophysiological mechanisms dependent on mitochondrial dysfunction in HF. Figure legend: Mitochondrial dysfunction is a typical feature of HF, being either a consequence or a cause of disease, and it leads to several molecular effects. The most relevant cellular pathways that are dysregulated in this condition, ending up as increased ROS level, are represented in the figure. The consequent cellular damage aggravates the disease contributing to HF progression. Abbreviations: ATP, adenosine triphosphate; DAMPs, damage-associated molecular patterns; ETC, electron transport chain; mPTP, mitochondrial permeability transition pore; NCLX, mitochondrial Na^+^/Ca^2+^ exchanger; OXPHOS, oxidative phosphorylation system; ROS, reactive oxygen species.

In this review, we will discuss the pathophysiological mechanisms, dependent on mitochondrial dysfunction, underlying HF development and progression as well as the potential implication of mitochondria as an attractive therapeutic target to treat and recover from HF (Figure 1).

## 2. Dysregulation of Fatty Acid Oxidation in HF

HF is characterized by a shift of energy production from mitochondrial fatty acid oxidation (FAO) to glycolytic pathways with the aim to maintain sufficient ATP levels [6,7]. However, this adaptive process does not represent an effective strategy to increase the energy supply since the ATP generated from glycolysis alone normally contributes to less than 5% of the total consumed ATP [8]. Accordingly, the hypothesis of energy starvation in HF suggests that mechanisms used to restore energy homeostasis might contribute to the vicious circle leading to cardiac remodeling and HF [9,10]. Indeed, increased glucose uptake and metabolism inhibit the branched-chain amino acid (BCAA) catabolism, promoting the mammalian target of rapamicine (mTOR) activation and cardiomyocyte hypertrophy [11]. Moreover, the inability to oxidize fatty acids could lead to the accumulation of lipotoxic metabolites [12]. In this regard, different studies demonstrated a dysregulation of several molecular mechanisms responsible for fatty acid metabolism. Evidence obtained in both animal models and humans showed that levels of peroxisome proliferator activated receptor-α (PPARα), a transcription factor responsible for fatty acid transport into the mitochondria and peroxisomes, and PPAR-γ co-activator (PGC)-1α are downregulated in HF [13,14,15]. On the other hand, malonyl-CoA levels are increased, resulting in the inhibition of carnitine O-palmitoyltransferase (CPT) 1 and mitochondrial fatty acid uptake [16]. Increased mitochondrial protein acetylation, including pyruvate and succinate dehydrogenases, malate-aspartate shuttle, tricarboxylic acid cycle, and fatty acid oxidation enzymes, has been described in HF models, also contributing to increased sensitivity to mitochondrial permeability transition pore (mPTP) opening [17,18,19]. It has been proposed that protein acetylation may be a consequence of an excessive concentration of short-chain acyl-CoA due to either reduced FAO or to decreased sirtuin-dependent protein deacetylation [20,21]. Indeed, sirtuin activity depends on NAD^+^ levels which are reduced in the presence of mitochondrial dysfunction and cardiomyocyte hypertrophy [22]. 

Other studies have shown a role of liver X receptor alpha (LXRα) deficiency in the development of impaired mitochondrial oxidative phosphorylation capacity. On the other hand, LXRα overexpression in mice resulted in increased glucose uptake with the increased expression of glucose transporter type 1 and 4 (GLUT1 and GLUT4) without significant effects on hexokinase 2, which catalyzes the phosphorylation of glucose; on phosphorylated adenosine monophosphate protein kinase (pAMPK), which regulates cardiac metabolism; and on CD36, the fatty acid transporter at the mitochondrial membrane [23]. 

The overexpression of adenine nucleotide translocase 1 (ANT1) has been proven to increase the activity of mitochondrial complexes II and IV and to decrease the release of caspase 3, the mPTP opening, and cellular apoptosis in different animal models [24]. 

Oxidative stress can also cause cardiomyocyte apoptosis by activating both the extrinsic pathway, through death receptor superfamily ligands, and the intrinsic pathway, through B-cell lymphoma 2 (Bcl-2) family proteins and mPTP opening [25]. Moreover, ROS-induced DNA damage may activate the transcription factor p53, which causes the translocation of Bcl-2-associated X protein and Bcl-2-associated death promoter to the mitochondria [26]. The overactivation of sympathetic system in HF also promotes norepinephrine-mediated tumor necrosis factor alpha (TNFα) secretion and the ROS-dependent activation of c-Jun N-terminal kinase (JNK) and p38-mitogen-activated protein kinase (MAPK) [27]. 

## 3. Hyperglycemia and Mitochondrial Dysfunction 

In diabetes, mitochondria switch the source of ATP production from glucose to fatty acid oxidation due to the lack or the insufficient action of insulin [28]. Hyperglycemia induces ROS production via the activation of NADPH oxidase, xanthine oxidase, and NO synthase, resulting in the disruption of the oxidative phosphorylation process [29]. Because of ROS damage, proteins and lipids are oxidized into reactive lipid peroxides and play a role in increasing the activity of uncoupling proteins (UCPs). The latter produce heat generation without ATP formation, resulting in impaired cellular insulin signaling [30]. 

The increased oxidative stress is associated with the generation of superoxides and hydrogen peroxide which contribute to cellular and mitochondrial damage in a vicious circle [31]. In addition, the imbalance of antioxidant defenses increases the susceptibility to oxidative damage, with the inactivation of the ETC complexes and mitochondrial proteins, and the impairment of the respiratory chain. In addition, the production of advanced glycation end products (AGEs) associated with hyperglycemia results in the AGE receptor (RAGE)-induced production of ROS and in the mPTP opening [31].

## 4. Mitochondrial Dysfunction and Ion Dynamics

Mitochondria can influence the Ca^2+^ dynamic since membrane-bound pumps, responsible for cytosolic Ca^2+^ release and removal, are energy-dependent [32].

A dysregulation of Ca^2+^ homeostasis occurs in HF, which consists in both an impaired reuptake by the sarcoplasmic reticulum and an increased leak through ryanodine receptors, contributing to mitochondrial dysfunction [33]. Indeed, Ca^2+^ regulates mPTP opening, mitochondrial membrane potential, ROS scavenging, and oxidative phosphorylation [34]. The deletion of the mitochondrial Na^+^/Ca^2+^ exchanger (NCLX), which is responsible for the efflux of Ca^2+^ into the cytosol, has been associated with mitochondrial Ca^2+^ overload, cellular necrosis, and sudden death in mice models [35]. However, the overexpression of NCLX does not improve mitochondrial function, suggesting that other mechanisms are involved in the regulation of Ca^2+^ levels [36]. 

With regard to mitochondrial Ca^2+^ uptake mediated by the Ca^2+^ uniporter (mtCU), the two regulators MICU1 and MICU2 were shown to be increased in failing human hearts compared to controls. This evidence suggested a possible role for adaptive/maladaptive changes in the mtCU composition and that the increase in the MICU1/MCU pore ratio correlated with a decrease in cardiac contractile function [37]. A dysfunction of mitochondria-associated endoplasmic reticulum membranes (MAMs) has also been described in HF, resulting in alterations in lipid and Ca^2+^ homeostasis, mitochondrial dynamics, and autophagy [38]. 

The dysfunction of ryanodine receptor 2 (RyR2) is also involved in the development of HF since it causes diastolic Ca^2+^ leaking, depleting sarcoplasmic reticulum stores and reducing cytoplasmic transients [39]. Both oxidative stress and hyperphosphorylation by protein kinase A and Ca^2+^/calmodulin-dependent protein kinase II (CaMKII) may contribute to RyR2 and Ca^2+^/ATPase (SERCA) alterations and Ca^2+^ overload [40]. Abnormal Ca^2+^ leaking may induce Na^+^ entry via the NCLX, resulting in the formation and propagation of delayed after-depolarizations and promoting ventricular arrhythmias [39,40]. ROS also promote the opening of L-type Ca^2+^ channels, thus inducing early after-depolarizations (EADs) [41]. ROS-derived fluctuations in the action potential cause areas of inhomogeneous excitability, which may trigger ventricular arrhythmias through re-entrant circuits [41]. Oxidative stress also contributes to interstitial fibrosis and alters the expression of connexin 43 (Cx43), the main component of cardiac gap junctions, further inducing arrhythmias [42]. 

In addition to the dysregulation of Ca^2+^ homeostasis, an impairment of K^+^ influx may occur in HF, resulting in mitochondrial depolarization and matrix swelling [43].

Mitochondrial K^+^ channels are involved in the regulation of energy production, Ca^2+^ retention capacity handling, membrane potential, and protection from ischemic/reperfusion injury [44]. Since most of these channels are opened by cyclic adenosine monophosphate (cAMP), cyclic guanosine monophosphate (cGMP), or both, the impaired mitochondrial bioenergetic associated with HF contributes to the dysregulation of K^+^ homeostasis in a bidirectional process [45]. 

A correct homeostasis of transition metals, such as iron, copper, and manganese, is fundamental for different metabolic pathways, including fatty acid oxidation, oxidative phosphorylation, the tricarboxylic acid (TCA) cycle, and glycolysis, which involve mitochondrial and non-mitochondrial enzymes such as electron transport chain (ETC) complexes, citrate synthase, ferro chelatase, aconitase, and xanthine oxidase [46]. 

ABC transporters in mitochondria regulate iron homeostasis and iron–sulfur cluster assembly. The deficiency of ABC transporters contributes to reduced mitochondrial ETC complex activity, iron overload, increased ROS production, and altered mitochondrial bioenergetics in HF [47]. 

Iron metabolism is regulated by two proteins (IRP1 and IRP2) which bind the iron response element (IRE) region of the transcripts of iron transporters such as ferroportin, transferrin, ferritin, and L-type calcium channels. IRP deficiency has been associated with impaired iron homeostasis, reduced mitochondrial performance, and increased ATP demand with an increased risk of HF after dobutamine challenge or MI in mice [48]. Metal manganese is an essential component of manganese-dependent superoxide dismutase (MnSOD/SOD2) and pyruvate carboxylase enzymes in mitochondria. It regulates Mg^2+^ and Ca^2+^ dependent mitochondrial enzymes [49]. Knock-out MnSOD mice show increased levels of mitochondrial ROS, complex I dysfunction and cardiomyocyte necrosis [50].

## 5. ROS-Induced Mitochondrial Damage

HF is associated with increased ROS production by components of the ETC at various sites within the inner mitochondrial membrane (IMM) and in the mitochondrial matrix through the Krebs cycle [9]. ROS may contribute to the damage of proteins and lipids, trigger the cell-death cascade, and compromise the cellular energy grid [51]. Aberrant mitochondrial membrane phospholipids, mainly consisting in cardiolipin decrements, play a role in the dysfunction of ETCs, mitochondrial ion homeostasis, and ROS production in HF [52]. Accordingly, the cell-permeable peptide MTP-131, a compound that targets cardiolipin in the mitochondria, was found to localize in the IMM and to improve bioenergetics by forming respiratory super-complexes [53]. Mitochondrial damage related to increased oxidative stress causes a further excessive production of ROS, resulting in a vicious circle of ROS-induced ROS release [54]. 

Under physiological conditions, superoxide is dismutated to hydrogen peroxide by superoxide dismutase, whereas hydrogen peroxide is removed by the antioxidant systems of peroxiredoxin (Prx) and glutathione peroxidase (Gpx) in the mitochondria [55]. In HF, an excessive mitochondrial ROS production occurs, contributing to cellular damage. A continuous reduction in thioredoxin 2 (Trx2) is necessary for the correct function of the mitochondrial Prx and Gpx systems and for the conversion of oxidized glutathione to glutathione using NADPH. Isocitrate dehydrogenase 2 (IDH2) and nicotinamide nucleotide transhydrogenase (Nnt) supply NADPH; thus, their dysfunction may play a role in the development and progression of HF [56]. However, other studies have shown that these enzymes divert NADH away from ATP production, resulting in a detrimental compensatory mechanism [57].

The phosphorylation of NAD^+^ by NAD kinase (NADK) in the cytosol (NADK1) and mitochondria (NADK2) is the only known mechanism by which NADPH is produced de novo. A reduced NADK activity has been documented in HF, contributing to the impaired capacity of detoxifying ROS [58]. 

## 6. ROS-Induced Mitochondrial Morphological and Functional Damage

The mitochondrial ROS level has been shown to be higher in peripheral blood mononuclear cells (PBMCs) from HF patients compared to controls both in the baseline condition and after lipopolysaccharide (LPS) and H_2_O_2_ stimulation. A parallel significant decrease in SOD and GPx activities was observed [59]. The cytofluorimetric analysis of mitochondrial membrane potential by tetramethylrhodamine methyl ester (TMRM) and JC-1 staining reflected a significant mitochondrial depolarization in HF subjects [59]. HF mitochondria showed significant ultrastructural changes with a reduced area carrying intact cristae and a convolution loss of IMM as detected by the IMM/outer MM (OMM) index [59]. When using the grading scale of mitochondrial damage (Mt-G), HF mitochondria showed a burden of overall damage, based on the distribution of Mt-G1 to Mt-G3 levels, significantly higher with respect to controls both at baseline and after LPS stimulation [59]. The flow cytometric analysis showed that the percentage of apoptotic cells in HF patients was significantly higher [59]. Transmission electron microscopy (TEM) revealed that the PBMCs of HF patients showed several features of cellular damage, such as apoptotic nuclei with areas of marginal, dense-stained chromatin, caryorrexis, caryolisis, fragmentation of cellular membranes, and a significant reduction in the mitochondrial area and perimeter, with a smaller total mitochondrial volume density. In addition, the mitophagic process (responsible for mitochondrial degradation), identified by the expression of Beclin 1, Parkin, and LC3, was downregulated in HF PBMCs [59]. 

Consistently with these results, other studies have shown an impaired morphology of mitochondrial cristae, with disorganization and reduced cristae density [60]. These structural changes were also detected in Parkin-knock-out cardiomyocytes which presented reduced mitophagy, altered cardiomyocyte size, and global cardiac structure with cardiac dysfunction [61]. The disruption of dynamic-related protein 1 (Drp1), which occurs in HF, results in the inhibition of mitophagy and contributes to cardiac dysfunction, leading also to increased susceptibility to ischemic/reperfusion injury [62]. Moreover, the impairment of mitophagy plays an important role in the development of diabetic cardiomyopathy favoring hypertrophy, diastolic dysfunction, and lipotoxicity [63]. 

## 7. ROS-Induced Inflammation and Cellular Damage

Because of ROS-induced damage, a leakage of mitochondrial DNA may occur, triggering inflammatory responses, stimulating the production of highly toxic peroxynitrate with a decrease in nitric oxide (NO) bioavailability and an increase in both hypoxia signaling and the MAP kinase pathway [64]. Different mechanisms are involved in the ROS-induced recruitment of circulating inflammatory cells and fibroblast progenitors including the secretion of chemokines, the activation of neutrophil integrins, and the expression of surface adhesion molecules by endothelial cells [65]. Moreover, the activation of the nuclear enzyme poly (ADP-ribose) polymerase stimulates the expression of inflammatory mediators and promotes the development of a subclinical inflammatory state which contributes to cardiac remodeling and HF progression [66]. HF is characterized by elevated levels of interleukin-6 (IL-6) and TNFα which play an important role in mitochondrial DNA damage, antioxidant factors’ inhibition, and ETC dysfunction with reduced ATP synthesis [32]. 

Excessive TNFα stimulation has been demonstrated to exert negative inotropic effects by reducing the release of Ca^2+^ from the sarcoplasmic reticulum, downregulating SERCA2a, uncoupling β-adrenergic receptors from adenylyl cyclase, inducing cardiomyocyte hypertrophy or cardiomyocyte apoptosis, and stimulating fibroblast proliferation and the secretion of MMPs [67]. The infusion of TNFα caused a progressive left ventricular (LV) dilatation and dysfunction [68]. Other studies have shown that the inhibition of TNFα improved the oxidative imbalance and reduced cellular apoptosis [69]. In addition to TNFα, elevated levels of IL-1b, IL-6, and IL-17 were described in patients with LV systolic dysfunction [70]. Accordingly, after the induction of an ischemic injury, a smaller infarct size with less fibrosis in the non-infarcted myocardium and reduced LV dilatation and systolic dysfunction have been reported in mice lacking IL-17 [71]. 

ROS-induced mitochondrial functional and morphological alterations contribute to myofibroblast differentiation and Smad signal transduction leading to fibrosis. In addition, ROS stimulate the TGF-β1 and NADPH oxidase 4 (NOX4) profibrotic signaling pathways in fibroblasts [72]. 

In such a context, it has been reported that the glucagon like peptide-1 receptor agonist (GLP1-RA) alogliptin alleviated interstitial fibrosis in diabetic rabbits by reducing the production of mitochondrial ROS and improving the swelling of mitochondria [73]. Also, mitoquinone (MitoQ), a mitochondrial-targeted antioxidant, has been proposed to inhibit fibrosis in pressure-overloaded hearts via targeting the above-mentioned biological pathways [74]. Similarly, ephedrine-4 could reduce cardiac fibrosis by maintaining the integrity of the mitochondrial membrane and preventing the release of cytochrome C53 [75].

Different studies proposed an initial protective role of inflammation during acute phases of cardiac damage such as ischemia–reperfusion injury through the upregulation of free radical scavengers and heat shock proteins [76]. Indeed, a larger LV infarct size has been documented in mice lacking TNFα receptors [77]. However, the persistence of inflammation becomes a maladaptive process which causes myocardial damage and contributes to HF pathophysiology. A continuous stimulation of toll-like receptors (TLRs) sustains chronic cardiac inflammation and damage [32]. Heat shock protein 60 (HSP60) has been found on the cell surface of cardiomyocytes from end-stage HF patients with a consequent activation of TLR4 [78]. Other studies have shown that the blockade of TLRs decreased inflammatory cytokine production and improved LV function after an ischemic injury [76,77]. 

In addition, the development of HF is favored by the release of damage-associated molecular patterns (DAMPs) derived from mitochondria, which are rich in unmethylated CpG motifs and N-formyl peptides that stimulate the NLRP3 inflammasome contributing to NAD^+^/NADH redox imbalance [79]. 

Regarding inflammatory cells, macrophages with an M1 phenotype as well as type 1 T-helper cells were associated with an inflammatory response, whereas an anti-inflammatory action of type 2 T-helper cells and T-regulatory cells has been described [80]. Indeed, fewer circulating T-regulatory cells have been documented [81]. This pro-inflammatory status is common also in HF with preserved ejection fraction (HFpEF), in which comorbidities such as obesity, diabetes, and hypertension promote the production of both cytokines (IL-1, IL-6, IL-23, TNFα, and TGFβ) and adipokines [82]. In the context of stressed adipose tissue, neutrophils and mast cells are attracted and promote the shift of quiescent macrophages to the M1 form. Adipokines, mainly leptin, stimulate the production of aldosterone and the activation of the sympathetic nervous system and neprilysin, with a consequent increased degradation of natriuretic peptides (NPs) and neurohormonal dysregulation [82]. A similar paracrine response at the epicardial fat tissue level also promotes microvascular rarefaction, fibrosis, and arrhythmogenesis [82]. Insulin resistance and hyperglycemia also contribute to inflammation through the increase of phosphoenolpyruvate carboxykinase, MAPK, extracellular signal-regulated kinase 1/1 and phosphoinositide 3-kinase activity, the reduction of adenosine monophosphate activated protein kinase, peroxisome proliferator activated receptor-γ and nuclear factor erythroid-related factor 2, the dysregulation of miRNAs and exosomes and the worsening of inflammatory and hypertrophic response and glucose metabolism [83]. Pressure overload in hypertensive patients has also been associated with the activation of the immune cascade of p38-MAPK, increased levels of cytokines, and the activation of TLRs and heat shock proteins [84]. Chronic kidney disease, another condition frequently coexistent with HF, is also related to a state of persistent subclinical inflammation, oxidative stress, and impairment of endothelial and vascular smooth muscle cell function [85].

## 8. Mitochondrial Dysfunction and Cardiomyocyte Hypertrophy

Dual-specificity tyrosine-regulated kinases (DYRKs) can reduce mitochondrial oxidative phosphorylation and activate Drp-1-mediated mitochondrial fission, accelerating cardiac hypertrophy and HF progression [86]. DYRK1B binds STAT3, inducing its phosphorylation, nuclear accumulation, and the downregulation of peroxisome proliferator-activated receptor γ coactivator-1α (PGC-1α) expression, finally resulting in impaired mitochondrial bioenergetics [87]. DYRK1B overexpression has been associated with a significantly increased weight-to-tibia length ratio, with larger heart size and cross-sectional area and with a thinner left ventricular wall [85]. Consistently, the mRNA expression of atrial natriuretic peptide (ANP), brain natriuretic peptide (BNP), and sarcomeric protein α-skeletal actin (Acta1), all markers of cardiac hypertrophy, were upregulated in DYRK1B transgenic hearts [87]. In addition, excessive fibrosis, consisting in extensive collagen deposition, increased collagen 1A1 mRNA expression, and reduced matrix metalloproteinase 9 (MPM9), was observed [87]. DYRK1B deletion restored cardiac performance, resulting in 28% and 38% increases in LV ejection fraction and LV fractional shortening, respectively [87]. DYRK1B overexpression also resulted in impaired mitochondrial morphology and density as detected by TEM. STAT3, an IL-6-activated transcription factor that plays a role in inflammation, cell growth, and metabolic regulation, was upregulated in the presence of DYRK1B-overexpression [88]. On the other hand, the expression of the transcriptional coactivator PGC-1α was significantly downregulated with a consequent impairment in mitochondrial bioenergetics [87].

The reduced expression in cardiomyocytes of Ndufc2, a subunit of mitochondrial complex I, has been associated with cellular hypertrophy [89]. An increase in cell size was detected in Ndufc2 silenced H9c2 cells, with the parallel upregulation of the expression of known markers of hypertrophy such as ANP and β-myosin heavy chain (MHC) mRNA levels [87]. Ndufc2 knockdown impaired mitochondrial function by compromising the redox status and unbalancing the oxidized form of nicotinamide adenine nucleotide (NAD^+^) and its reduced form (NADH). A decrease in the NAD^+^/NADH ratio was detected in parallel with reduced levels of sirtuin 3 (SIRT3) and MnSOD, decreased activation of AMPK, and increased phosphoAKT and ROS levels in Ndufc2 knock-out cells [89]. The supplementation of NAD^+^ through nicotinamide reduced hypertrophy, as documented by the decrease in cell size and the hypertrophy markers, and it was associated with an improvement in the mitochondrial membrane potential and a reduction in intracellular ROS [89]. In hypertensive patients, the TT genotype at *NDUFC2/rs11237379*, associated with significantly reduced gene expression [90], was associated with LV hypertrophy, with a significant increase in septal thickness, posterior wall thickness, relative wall thickness (RWT), and LV mass/BSA compared to subjects carrying either CC or CT genotypes. Patients carrying the A allele at *NDUFC2/rs641836* also showed a significant increase in septal thickness, posterior wall thickness, LV mass, LV mass/BSA, and LV mass/height^2.7^ [89]. It is likely that hypertensive patients carrying the *NDUFC2* gene variants may have an increased occurrence of HF, although studies are still lacking in this regard.

Consistently, different studies have demonstrated the protective mechanisms of sirtuins. Indeed, sirtuins activate the pAMPKα pathway, increase Bcl2 protein levels, inhibit NF-κB, decrease phosphoprotein kinase B protein, regulate energy metabolism through acetylation, suppress Wnt3a expression, and reduce Caspase3 mRNA and PARP1 protein levels. As a result, sirtuins exert protective effects against hypoxia-induced mitochondrial dysfunction, cytosolic release of cytochrome C, cardiac hypertrophy, and HF development [91]. 

ROS have been involved in different signaling pathways leading to cardiomyocyte hypertrophy. In HF, neurohormonal dysregulation with angiotensin II, endothelin-1, and phenylephrine overproduction stimulates ventricular cardiomyocyte hypertrophy through the redox-dependent activation of apoptosis signal-regulating kinase 1 (ASK1) and NF-kB [92]. The renin-angiotensin system also activates nicotinamide adenine dinucleotide phosphate oxidases (Nox) which induce cellular hypertrophy regardless of blood pressure levels. Nox activity is stimulated under pressure overload conditions and activates ROS-dependent extracellular signal-regulated kinases 1/2 [93]. Nox2-derived ROS activate the JNK/nuclear factor of the activated T-cell signaling pathway, inducing the differentiation and proliferation of cardiac fibroblasts [94]. Nox2 has been also demonstrated to induce the upregulation of connective tissue growth factor (CTGF), NF-kB, and matrix metalloproteinases (MMPs), causing an excessive collagen deposition and a consequent structural modification of the extracellular myocardial matrix [95]. After the exposure to angiotensin II and aldosterone, fibrosis was significantly reduced in Nox2-null mice compared to wild-types [96]. 

## 9. Mitochondria as Potential Therapeutic Targets in HF

According to the abovementioned evidence, mitochondria may represent a suitable therapeutic target in HF [97]. 

Effective therapies may include fatty acid (FA) metabolic regulators, glucose metabolic modulators, mitochondrial OXPHOS regulators, antioxidants, and mitochondrial quality control regulators [98,99].

FA metabolism may be regulated by PPARα agonists and L-Carnitine. Fibrates, activating PPARα, have shown promising evidence in HF by improving LV function, preventing myocardial fibrosis, and improving diastolic function [100]. 

In an animal model of HFpEF, L-Carnitine treatment has been shown to restore LV free-carnitine levels, to attenuate LV fibrosis and stiffening, and to improve survival. In cultured cardiac fibroblasts, L-Carnitine reduced angiotensin II-induced collagen production [101]. Another study conducted in 246 patients with HF due to coronary artery disease showed that L-Carnitine improved LV function [102]. 

Besides their role in regulating glucose metabolism, SGLT2i have been shown to increase FA oxidation and ketogenesis and to rebalance the relationship between glycolysis and OXPHOS. These biological properties may contribute to explaining the cardioprotective effects of SGLT2i in HF (reduction in cardiovascular mortality and HF hospitalizations across the entire LVEF) [103].

An increasing body of evidence has suggested a potential therapeutic role of the stimulation of mitochondrial biogenesis through the activation of the AMPK and NO/soluble guanylyl cyclase (sGC)/cGMP pathways [99]. Different drugs with cardioprotective effects such as metformin, thiazolidinediones, and statins indirectly activate AMPK [99]. Direct AMPK activators, including 5-aminoimidazole-4-carboxamide riboside (AICAR), A-769662, and PT-1, are currently under various stages of development [104]. 

Based on previous evidence that abnormal mitochondrial structure and function are associated with altered mitophagy and increased oxidative stress in HF with reduced ejection fraction (HFrEF) [59], a recent study tested the impact of ANP (able to stimulate autophagy/mitophagy in cardiomyocytes [105]) in HFrEF patients by both ex vivo and in vivo approaches [106]. In the ex vivo study, PBMCs isolated from HFrEF patients were directly exposed to αANP. The in vivo study included HFrEF patients who received a treatment with sacubitril/valsartan, a first-line pharmacological therapy consisting in the association of the type 1 angiotensin II receptor and neprilysin inhibitors, the latter causing an increase in NPs, mainly αANP [106]. Both the ex vivo direct exposure to αANP and the higher αANP level upon in vivo treatment were able to restore mitochondrial membrane potential, to stimulate the autophagic process with an increase in the mitochondrial mass index, to reduce mitochondrial damage with an increased IMM/OMM index, and to decrease ROS levels [106]. According to these results, the favorable effects of NPs on mitochondrial function may contribute to explain, at least in part, the efficacy of sacubitril/valsartan in reducing cardiovascular mortality and HF hospitalizations and their role as a first-line strategy in the therapeutic management of HF. 

With regard to antioxidants and mitochondrial quality control regulators, different studies have shown a potential role of CoQ10 [107]. In a zebrafish model, the depletion of UBIAD1, a nonmitochondrial CoQ10-forming enzyme, resulted in increased oxidative stress and cardiac damage. In animal models of isoproterenol-induced HF, CoQ10 levels were significantly reduced and their supplementation improved LV function [108]. In a mouse model of diabetic cardiomyopathy, CoQ10 supplementation reduced cardiomyocyte hypertrophy and fibrosis and improved LV diastolic function [109]. 

Different studies have investigated the role of CoQ10 supplementation in patients with HF with controversial results. The myocardial CoQ10 levels were inversely related to the NYHA class in 43 HF patients. In the Controlled Rosuvastatin Multinational Study in HF (CORONA), patients in the lowest tertile of CoQ10 had significantly lower LVEF and higher NT-proBNP levels [110]. However, elevated CoQ10 levels were associated with an increased risk of mortality in 236 patients hospitalized for HF [111]. A meta-analysis which included 395 patients from 13 studies showed a 4% improvement in LVEF in those who received CoQ10 compared to a placebo [112]. A study enrolling 914 patients did not show any significant improvement in LVEF or exercise capacity after treatment with CoQ10 [113]. The Q-SYMBIO (Coenzyme Q10 as Adjunctive Treatment of Chronic Heart Failure: A Randomised, Double-blind, Multicentre Trial With Focus on Symptoms, Biomarker Status) study, which enrolled 420 patients, demonstrated that CoQ10 reduced the composite primary endpoint of cardiovascular death, hospital stays for HF and mechanical support or cardiac transplant, as well as the secondary outcomes of death from cardiovascular causes and all-cause mortality, compared to the placebo [114]. However, the study had significant limitations such as the incomplete enrollment that would have required an 8-year period, the small number of events, and the limited sample size [107]. In patients with ischemic heart disease, CoQ10 at 300 mg/day for 3 months demonstrated a significant reduction in inflammatory markers, such as TNFα and IL-6, compared with a placebo [115]. 

In a mouse model of HFpEF, myocardial fibrosis was reduced by the inhibition of the NLPR3 inflammasomes with histone deacetylation and improvement in mitochondrial hyperacetylation and dysfunction [116].

Another potential mitochondrial target is represented by the mitochondrial pyruvate carrier (MPC), that mediates the import of pyruvate into the mitochondrial matrix across the mitochondrial inner membrane. Thus, MPC blockers might restore mitochondrial dysfunction by modulating the oxidative phosphorylation system [117]. 

Mitochondrial function might be also regulated by inhibitors of mPTP such as cyclosporine A and by lipid-binding molecules such as the cardiolipin (CL)-binding peptide elamipretide [97]. In rats with myocardial injury, the chronic administration of elamipretide significantly reduced ROS production and cytosolic cytochrome c levels in the peri-infarcted region restoring cardiac function [118]. 

Sonlicromanol, a new molecule acting as redox modulator (via mPGES-1 inhibition) and as an antioxidant (via the thioredoxin/peroxiredoxin system), has been tested in patients with mitochondrial diseases demonstrating a good safety and tolerability profile without significant cardiovascular adverse events [119]. 

Another molecule targeting mitochondrial ROS production is OP2113 [5-(4-methoxyphenyl) dithiole-3-thione], also known as anethole trithione or Sulfarlem. It has been shown that OP2113 inhibits up to 80% of superoxide and hydrogen peroxide production by respiratory complex I (IQ site) [120]. However, no clinical trials have specifically investigated the efficacy of OP2113 in HF.

In spontaneously hypertensive rats, the pharmacological inhibition of poly(ADP-ribose) polymerase (PARP) improved mitochondrial morphology by reducing mitochondrial fragmentation and increasing mitochondria size and cristae density, thus resulting in the prevention of LV hypertrophy [121]. In mice with pressure overload-induced HF, treatment with the mitochondrial division inhibitor (Mdivi) and with berberine, a substance able to activate mitophagy via the PINK1/Parkin pathway, decreased ventricular fibrosis and preserved cardiac function [122,123]. 

Other promising pharmacological classes are represented by agents interacting with the respiratory chain components. In such a context, idebenone is a synthetic short-chain analogue of coenzyme Q10 with improved solubility and pharmacokinetics [124]. Also, imeglimin, a new antidiabetic drug that amplifies stimulated insulin secretion (GSIS) and enhances insulin action, has been observed to rebalance respiratory chain activity by correcting the deficiency of Complex III activity and preventing mPTP opening [125]. 

## 10. Conclusions

Mitochondrial dysfunction is a feature of HF, being either a consequence or a cause of disease. Therefore, HF shows a maladaptive response, a progressive decline in bioenergetic reserve capacity, a shift of energy production from mitochondrial fatty acid oxidation to glycolytic pathways, a dysregulation of ion homeostasis, and an increased production of ROS. Oxidative stress is related to morphological and functional dysfunction which contributes to the development of cardiac hypertrophy and to the progression of HF. 

Although further studies are needed to confirm and extend the available evidence, a promising role of mitochondria as a therapeutic target in HF is emerging and may pave the wave for future effective strategies in the management of this condition. 

## Data Availability

Not applicable.
